# Diet-Intestinal Microbiota Axis in Osteoarthritis: A Possible Role

**DOI:** 10.1155/2016/3495173

**Published:** 2016-08-17

**Authors:** Yusheng Li, Wei Luo, Zhenhan Deng, Guanghua Lei

**Affiliations:** Department of Orthopaedics, Xiangya Hospital, Central South University, Changsha, Hunan 410078, China

## Abstract

Intestinal microbiota is highly involved in host physiology and pathology through activity of the microbiome and its metabolic products. Osteoarthritis (OA) is a common form of arthritis characterized by articular cartilage destruction and osteophyte formation. Although various person-level risk factors, such as age, sex, and obesity, have been proposed for the pathogenesis of OA, the underlying links between these person-level factors and OA are still enigmatic. Based on the current understanding in the crosstalk between intestinal microbiota and these risk factors, intestinal microbiota could be considered as a major hidden risk factor that provides a unifying mechanism to explain the involvement of these person-level risk factors in OA.

## 1. Intestinal Microbiota

All metazoan, from invertebrates to vertebrates, have intestinal microbiota. Intestinal microbiota is highly associated with various aspects of metazoan physiology, such as development, metabolism, and immunity, and is decidedly involved in the pathogenesis of various metazoan diseases, such as inflammatory bowel disease, obesity, and cancer [1, 2]. Even for some invertebrates that have a relative simple of intestinal microbiota, such as* Caenorhabditis elegans *(one bacterial species) and* Drosophila melanogaster *(5–20 species), the intestinal microbiota still has profound influence in host physiology, like host longevity [1]. The influence of gut microbiota on host physiological functions and pathogenesis of diseases may result from activity of the microbiome and its metabolic products [1]. Although these years have witnessed a compelling progression on the involvement of dysbiosis of intestinal microbiota in rheumatoid arthritis [3], the roles of intestinal microbiota or its metabolic products in the pathogenesis of osteoarthritis (OA) remain to be known.

OA is one of the most common joint disorders worldwide. The exact etiology of OA is still unknown, but various risk factors have been reported, including person-level factors, such as age, sex, obesity, and diet, and joint-level factors, including injury, malalignment, and abnormal loading of the joints [4–6]. Regarding person-level factors, a number of explanations have been proposed to explain the involvement of person-level factors in OA; however, most of these hypotheses have not been demonstrated experimentally, and even some have been challenged by later observational studies and clinical trials. In this paper, we try to explain the involvement of some person-level factors in OA from aspect of intestinal microbiota and try to highlight the importance of intestinal microbiota or its metabolic products in the pathogenesis of OA.

## 2. Age and OA

Age is regarded as the main predictor of OA [6]; however, the underlying mechanisms behind the increased prevalence and incidence of OA with age remain to be known. Although a number of explanations, including oxidative damage, thinning of cartilage, muscle weakening, and a reduction in proprioception, have been proposed to explain the involvement of age in OA, none of these hypotheses have been demonstrated empirically.

With cell culture-dependent and culture-independent studies, older people show significant difference in gut microbiota from that of younger adults, such as lower diversity of gut microbiota, greater proportion of* Bacteroides* spp., and distinct abundance pattern of* Clostridium* groups [7, 8]. The alteration of gut microbiota may regulate the age-related physiology, such as immune responses, cognitive function, and organ disorders [7, 8]. For example, dysbiosis of intestinal microbiota highly precedes and predicts age-onset intestinal barrier dysfunction in* Drosophila*, while targeting of the aging-related dysbiosis of intestinal microbiota alleviates these age-related dysfunctions and increases life span in* Drosophila *[9, 10]. Thus, it is utterly possible that the aging-related dysbiosis of intestinal microbiota contributes to age as a risk factor for OA.

## 3. Gender and OA

It is an interesting observation that the prevalence and severity of OA in hip, knee, and hand are higher in women than in men, and they also increase around menopause [11]. For example, with one study with 4733 subjects in Alberta, Canada, the prevalence of knee OA is 6.3% for males and 8.9% for females, while the prevalence of hip OA is 4.4% for males and 7.6% for females [12]. This discovery suggests presence of sex difference in OA prevalence and incidence, with females generally at a higher risk. This compelling investigation results in a hypothesis that hormonal factors, like oestrogen, have role in the development of OA. However, later conclusions from observational studies and clinical trials challenge this hypothesis [13, 14], and other explanations have been proposed for the discrepancy between men and women in OA, such as differences in volume of cartilage, bone strength, and muscle strength.

Male mice have more bacteria overall in the feces compared to female mice; however, female mice show more significant diurnal oscillation in intestinal microbiota than that of male mice [15], indicating that gender has remarkable influence on intestinal microbiota. Indeed, gender-dependent influence in intestinal microbiota has been observed in various animal models [16], such as fish, mice, and human. For example, with 16S DNA sequencing of intestinal microbiota in fecal samples of 39 men and 36 postmenopausal women with similar dietary background and age, the abundance of* Bacteroides* genus and the abundance of* Bilophila* are lower in men than in women, while higher presence of* Veillonella* and* Methanobrevibacter* genera is observed in men compared to women [17]. Thus, the difference in intestinal microbiota may be responsible for the higher risk of OA in women. Similarly, female mice have higher incidence (1.3–4.4 times) of type 1 diabetes (T1D), compared to male mice [18, 19]. Similar to estrogen therapy which has little effect on OA [13, 14], the protection of males against T1D does not correlate with blood androgen concentration [18]. However, germ-free (GF) mice lack the gender bias, while colonization of GF mice with some lineages of overrepresented microbiota in male mice (i.e.,* Enterobacteriaceae* family) restores the gender bias for T1D [18]. Interestingly, transplantation of gut microbiota from adult males to immature females alters the recipient's microbiota, leading to elevated testosterone, reduced islet inflammation and autoantibody production, and finally increased T1D protection [19]. These results show that intestinal microbiota regulates disease fate in individuals; thus it is possible that intestinal microbiota contributes to gender as a risk factor for OA.

## 4. Obesity and OA

One of the well-known risk factors for OA is obesity [20, 21]. C57BL/6J male mice fed a high-fat diet (HFD) for 12 weeks have greater body weight and also exhibit features consistent with knee OA, compared to the control mice [22]. With 1,764,061 observed subjects, for a median (interquartile range) of 4.45 (4.19 to 4.98) years, overweight or obesity increases the risk of OA at knee, hip, and hand, especially at the knee: overweight and (grades I and II) obesity increase knee OA risk 2-, 3.1-, and 4.7-fold, respectively [23]. With one study with 4733 subjects in Alberta, Canada, obesity (BMI > 30 kg/m^2^) is remarkably associated with the prevalence of knee and hip OA [12]. However, the mechanism by which obesity boosts OA is enigmatic. The link between obesity and OA was contributed to excessive joint loading as a result of increased body weight; however, a more complex aetiology for obesity-induced OA has been indicated, such as disturbed lipid metabolism, low-grade inflammation, and adipokines on joint tissues.

It is well known that intestinal microbiota is associated with the establishment and development of obesity. Obesity is associated with phylum-level changes in the microbiota (i.e., ratio of Firmicutes/Bacteroidetes), reduced bacterial diversity, and altered representation of bacterial genes and metabolic pathways [24, 25]. In genetically obese* ob/ob *mice and obese people, ratio of Firmicutes to Bacteroides is augmented, which promotes production of biologically active metabolites, such as short chain fatty acids (SCFAs), including acetate, propionate, and butyrate, from soluble dietary fibers (i.e., fructans) and resistant starch, leading to higher energy extraction from indigestible carbohydrates and to adipogenesis in the liver [24, 25]. As the dysbiosis of intestinal microbiota contributes to obesity and to other obesity-related conditions including insulin resistance and systemic inflammation, it is reasonable that intestinal microbiota is associated with pathogenesis of OA.

## 5. Diet and OA

Several dietary factors have been reported to be involved in pathogenesis of OA, such as vitamins [26], amino acids [5], and magnesium [27]. However, further studies are needed to better define the association between OA and these dietary factors and to better understand the underlying mechanism for these dietary factors to regulate OA. Intestinal microbiota is highly shaped by dietary nutrients [7, 28, 29]. For example, little amount of single amino acids supplementation (0.5% (w/w) L-arginine or 1.0% (w/w) L-glutamine) has shown significant influence on intestinal microbiota, such as the ratio of Firmicutes/Bacteroidetes [28, 29]. The possible reason for nutrient-induced change of intestinal microbiota is that nutrient alters the microenvironment for intestinal microbiota, such as composition and metabolism of intestinal microbiota, and immune responses of host. Glutamine supplementation promotes mouse intestinal secretory IgA (SIgA) production and IgA^+^ plasma cell numbers through T cell-dependent (e.g., IL-5, IL-6, and IL-13) and T cell-independent pathways [e.g., transforming growth factor (TGF-*β*), a proliferation-inducing ligand (APRIL), and B cell-activating factor (BAFF)] of SIgA induction, which are largely dependent on glutamine's effect on intestinal microbiota [30]. Dietary chitosan supplementation significantly shapes the intestinal microbiota in mouse model [31]. Although chitosan has little effect on the richness indices of intestinal microbiota in mouse jejunum, ileum, and feces, it highly affects the microbiota diversity in the jejunum, ileum, and feces of mouse [31]. Chitosan also alters the component of intestinal microbiota, including lowering the ratio of Firmicutes : Bacteroidetes, decreasing the Bacteroidales in the feces, and increasing the Lactobacillalesin the feces [31]. Indeed, chitosan supplementation decreases mouse body weight through its effect on intestinal microbiota [31]. These interesting investigations indicate that nutrient affects host physiological functions largely dependent on intestinal microbiota. Thus, the nutrients-intestinal microbiota axis may be implicated in pathogenesis of OA. If so, the manipulation of nutrients-intestinal microbiota axis is auspicious to prevent and treat OA. Indeed, oral supplementation of resveratrol has significant anti-OA effects in HFD-induced OA model in mouse through recovery in joint structure and type II collagen expression in cartilage and inhibition in the degradation of type II collagen into C-telopeptide of type II collagen (CTX-II) and chondrocyte apoptosis [22].

## 6. Conclusion

**Figure 1:**
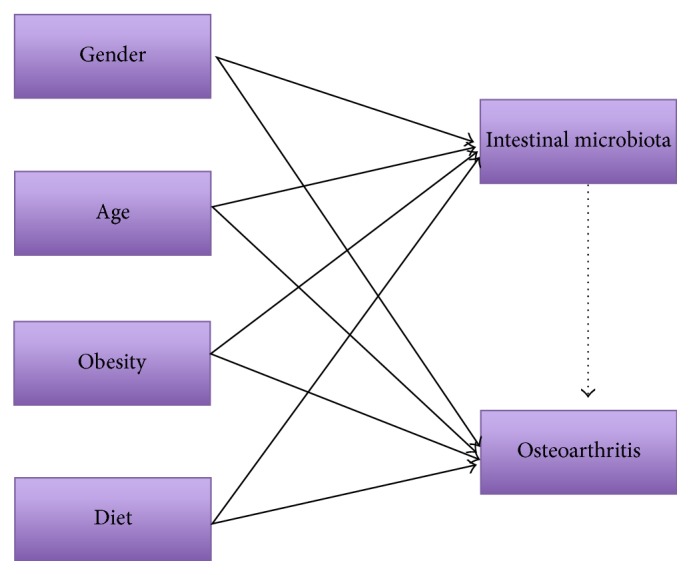
Intestinal microbiota in osteoarthritis (OA). Age, sex, obesity, and diet are risk factors in the etiology of OA (full line) and are associated with the alteration of intestinal microbiota (full line). Thus, it is possible that the intestinal microbiota is involved in the pathogenesis of OA (dotted line).

The intestinal microbiota is profoundly associated with pathogenesis of various diseases, such as inflammatory bowel disease, obesity, and cancer [1, 2]. Here we describe the rationale for the hypothesis that intestinal microbiota is a major hidden risk factor for OA and an important explanation for person-level risk factors in OA (Figure 1). Although bacterial lipopolysaccharide has been suggested as hidden risk factor for OA [32], it is fruitful to explore the OA patient related changes in microbiota composition, bacterial diversity and bacterial genes, and metabolic pathway. The understanding on the association between intestinal microbiota and OA could facilitate the development of new approaches to diagnosing and treating OA. In particular, with the knowledge of intestinal microbiota in pathogenesis of OA, the manipulation of nutrient-intestinal microbiota- bacterial metabolite axis has potentials to prevent and treat OA.
